# Comparison of Robot-Assisted Laparoscopic Partial Nephrectomy with Laparoscopic Cryoablation in the Treatment of Localised Renal Tumours: A Propensity Score-Matched Comparison of Long-Term Outcomes

**DOI:** 10.3390/diagnostics11050759

**Published:** 2021-04-23

**Authors:** Hui-Ying Liu, Chih-Hsiung Kang, Hung-Jen Wang, Chien-Hsu Chen, Hao-Lun Luo, Yen-Ta Chen, Yuan-Tso Cheng, Po-Hui Chiang

**Affiliations:** 1 Department of Urology, Kaohsiung Chang Gung Memorial Hospital and Chang Gung University College of Medicine, Kaohsiung 83301, Taiwan; ying1011@cgmh.org.tw (H.-Y.L.); chkang5801@gmail.com (C.-H.K.); hujewang@gmail.com (H.-J.W.); u8601062@cgmh.org.tw (C.-H.C.); alesy@cgmh.org.tw (H.-L.L.); adam@cgmh.org.tw (Y.-T.C.); ytcheng@cgmh.org.tw (Y.-T.C.); 2 Graduate Institute of Medicine, College of Medicine, Kaohsiung Medical University, Kaohsiung 83301, Taiwan

**Keywords:** laparoscopic cryoablation, localised renal tumours, robot-assisted laparoscopic partial nephrectomy

## Abstract

Preserving renal function and controlling oncological outcomes are pertinent when managing renal neoplasms. Cryoablation is the recommended treatment only for clinical T1a stage renal tumour. Here, we compared the outcomes of robot-assisted laparoscopic partial nephrectomy (RaPN) and laparoscopic cryoablation (LCA) in the treatment of patients with localised T1-T2 renal tumours. Overall, 86 patients who received RaPN and 78 patients underwent LCA were included in this study. The intraoperative, postoperative, and oncological outcomes in the LCA group were non-inferior to the RaPN group. Moreover, LCA demonstrated shorter operative time (267.45 ± 104.53 min vs. 138.56 ± 45.28 min, *p* < 0.001), lower blood loss (300.56 ± 360.73 mL vs. 30.73 ± 50.31 mL, *p* < 0.001), and slight renal function deterioration because of the reduced invasiveness, without compromising on the oncological outcomes.

## 1. Introduction

The increased awareness and application of high-resolution imaging techniques have led to higher diagnosis rates of renal masses in recent decades [1,2]. Small renal mass (SRM) incidence increases by 2–3% annually [3]; approximately 20% of the SRMs are benign neoplasms [3,4]. When making treatment decisions, general kidney function preservation and perioperative outcomes should be considered in conjunction with oncological outcomes [5]. Consequently, nephron-sparing surgery and ablative techniques have gained interest worldwide [6].

Robot-assisted laparoscopic partial nephrectomy (RaPN), a minimally invasive nephron-sparing surgery, preserves renal function better than does radical nephrectomy [5], and significantly reduces complication rates compared with open partial nephrectomy (OPN) [7]. Ablative therapies have emerged as an alternative to partial nephrectomy, particularly in patients of advanced age, short life expectancy, or high comorbidity rates [1,3]. Laparoscopic cryoablation (LCA), another minimally invasive technique, does not impose surgical ischaemia on the kidneys because of its in situ ablative nature [6].

We have been performing LCA since 2008 and RaPN since 2015 at our hospital. This study compared the perioperative, functional, and oncological outcomes of these two treatment modalities in patients with T1-T2 localised renal tumours.

## 2. Materials and Methods

### 2.1. Study Population

**Figure 1:**
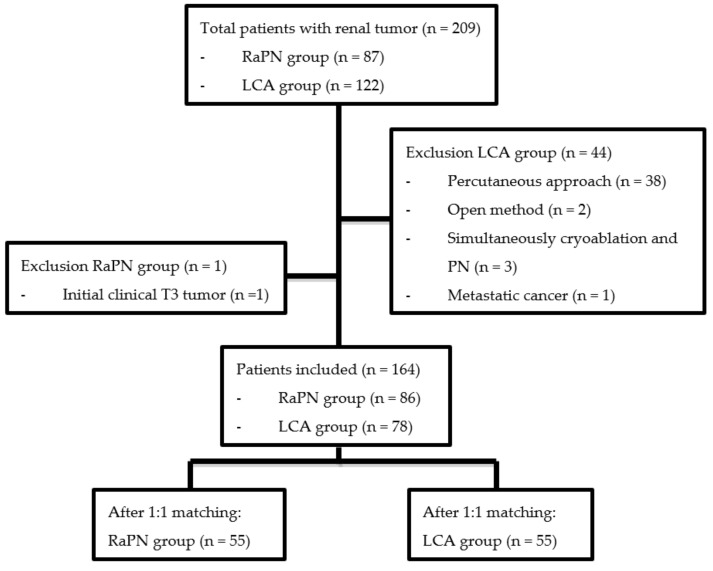
Flow chart for patients’ selection.

We retrospectively collected our hospital data of a cohort of patients diagnosed as having a localised T1-T2 renal tumour and treated using either RaPN or LCA. In our hospital, we began using the da Vinci robotic platform (“Intuitive Surgical” Instrument Control System, Endoscopic Instruments and Accessories, Intuitive Surgical, Inc., Sunnyvale, CA, USA) for RaPN in April 2015, whereas the application of cryoablation therapy for renal tumour management through open method, ultrasonography or computed tomography (CT) guidance percutaneous method, or laparoscopic method was initiated in July 2008. Patient selection was based on the European Association of Urology guidelines and the TNM classification for localised renal tumours. To minimise the difference among cryoablation groups, we only compared LCA with RaPN. Patients at initial clinical stage T3 or with metastatic cancers were excluded. The recruited patients were followed until 2021. In total, 164 patients (86 receiving RaPN and 78 receiving LCA) were recruited. Propensity score matching was performed in a 1:1 nearest neighbour manner by using gender, age, American Society of Anesthesiologists (ASA) score, body mass index (BMI), comorbidities, tumour side, tumour size, clinical T stage, and RENAL nephrometry score to generate the conditional treatment probability. These variables were selected to reduce the differences in baseline characteristics between cryotherapy and partial nephrectomy (PN). Finally, 55 patients who received RaPN and 55 patients who received LCA were analysed. The patient selection flow is illustrated in Figure 1.

### 2.2. Preoperative Assessment and Perioperative Data

Baseline parameters, including gender, systemic diseases, age, ASA score, BMI, haemoglobin and haematocrit levels, anaesthesia type, tumour size, and cryoablation probe numbers, were recorded. Preoperative assessment, including blood testing, clinical evaluation, and staging imaging evaluation, was used to define the general performance status of the patients and assess their suitability for interventional procedures. The RENAL nephrometry scores were assessed to distinguish the complexity of renal tumours in both groups [3,8].

Intraoperative time, bleeding, length of stay (LOS), follow-up duration, pathological diagnosis, and oncological outcome were reported in the outcome data. The early postoperative complications (up to day 30 postoperatively) were recorded and assessed according to the Clavien–Dindo classification system [9]. Renal function was recorded preoperatively and followed up 1 day, 3 months, and annually postoperatively. The estimated glomerular filtration rate (eGFR) was calculated using the Modification of Diet in Renal Disease (MDRD) GFR equation [6].

### 2.3. Statistical Analyses

Arithmetic values are expressed as mean ± standard deviation. Preoperative and postoperative continuous variables were compared using the independent sample *t* test and Mann–Whitney U test. Categorical data were analysed using Pearson’s Chi-square test and Fisher’s exact test. Renal function was analysed using a generalised linear model. A *p* value of <0.05 was considered statistically significant. All statistical analyses were performed using SPSS (version 20; SPSS Inc., Chicago, IL, USA).

### 2.4. Surgical Techniques: RaPN

The RaPN procedure was performed under general anaesthesia in the lateral position. In most patients, four ports (three robotic and one assistant port) were placed using either a retroperitoneal or transperitoneal approach according to the tumour location. After dissecting the renal pedicles and identifying the tumour, the resection margins were marked with cautery under intraoperative ultrasound guidance. A bulldog clamp is typically applied on the renal artery. Complete excision of the tumour and part of the surrounding normal kidney tissue allowed a safety margin and ensured efficient reconstruction. The resulting defect was closed in one or two layers. The bulldog clamp was then removed. The excised tumour was then trapped in a retrieval bag and delivered through the assistant port.

### 2.5. Surgical Techniques: LCA

All patients underwent LCA under general anaesthesia in an overextended flank position to allow a retroperitoneal or transperitoneal approach according to the tumour location and doctor’s preference. Most of the doctors prefer a retroperitoneal approach for posterior tumour, and transperitoneal approach for anterior tumour. After renal mobilisation, an 18-gauge core biopsy instrument was used on the renal tumour under the guidance of intracorporeal colour Doppler ultrasound. Then, one to six cryoprobes (the manufacturers of cryoprobe are ENDOCARE, INC., IRVINE CA, USA and Galil Medical, Yokneam, Israel) were inserted to perform a double-freezing cycle with argon, followed by an active thaw cycle with helium, under the guidance of intracorporeal ultrasound, to identify the definite tumour location and monitor ice-ball coverage. After completing two freeze–thaw cycles, the probes were removed, and haemostasis was achieved with cautery or the use of gelatin–thrombin matrix product (FloSeal, Baxter, Hayward, CA, USA) to the renal surface.

## 3. Results

The propensity score-matched study population comprised 55 patients who underwent RaPN and 55 patients who underwent LCA. The demographic characteristics were similar in both groups (Table 1). The tumour characteristics expressed with the RENAL nephrometric scores indicated no differences between the two groups (Table S1).

### 3.1. Perioperative Data and Pathological Outcome

The intraoperative and postoperative outcomes are presented in Table 2. We began applying LCA in our hospital before RaPN. The mean follow-up time was longer in the LCA group than in the RaPN group (54.96 ± 34.59 vs. 33.20 ± 19.55 months, *p* < 0.001). The LCA group had a shorter operative time (*p* < 0.001) than the RaPN group did. The estimated blood loss was significantly higher in the RaPN group than in the LCA group (*p* < 0.001). No significant differences were noted in complications; however, more fever episodes were observed in the RaPN group than in the LCA group (*p* = 0.012). The postoperative complications, according to the Clavien–Dindo classification system, were recorded. Most complications were temporary and self-resolving. One patient in the RaPN group demonstrated colon laceration and performed primary suture repair, another patient converted to open method. One patient in the LCA group discovered pneumothorax and subcutaneous emphysema after operation required pigtail catheter drainage, another patient demonstrated delayed haemorrhage, which required a transcatheter arterial embolism to control the bleeding. There was no significant difference in the complication rates between the two groups.

Among pathological outcomes, different renal cell carcinoma (RCC) types and benign lesions were recorded separately (Table 3). In total, 32 (58.2%) and 27 (49.1%) patients were diagnosed as having RCC in the RaPN and LCA groups, respectively (*p* = 0.339). No significant difference was observed in the number of patients with different types of RCC or benign conditions. However, data were more inconclusive in the LCA group, owing to the small amount of tissue being obtained during biopsy than during tumour resection in the RaPN group.

### 3.2. Oncological Outcomes

In patients with RCC sized ≤4 cm, the oncological outcomes were favourable. No patient demonstrated local recurrence, metastasis, or de novo tumour in both groups (Table 4). One patient, a hepatitis B virus (HBV) carrier, died of HBV flare up and hepatic failure in the RaPN group; another patient with a history of hepatocellular carcinoma (HCC) died of spontaneous bacterial peritonitis in the LCA group. By contrast, in patients with RCC sized >4 cm, oncological outcomes were acceptable. Two patients in the LCA group demonstrated local recurrence, and one patient demonstrated metastasis with a de novo tumour in the RaPN group (Table 5). One patient with a history of HCC died of spontaneous bacterial peritonitis and hepatic encephalopathy in the LCA group. None of the patients died from RCC either tumour size ≤4 cm or >4 cm during the follow-up period. The overall survival rate in the patients with RCC either tumour size ≤4 cm or >4 cm is shown in Figures S1 and S2.

### 3.3. Renal Function Outcomes

**Figure 2:**
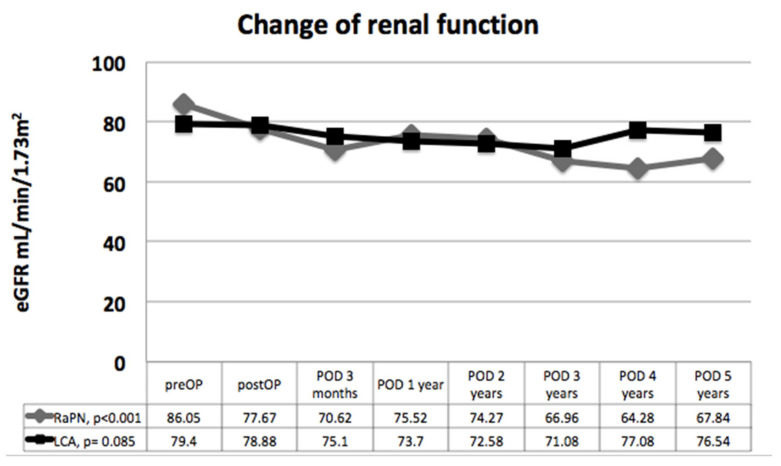
Change of renal function outcomes with estimated glomerular filtration rate (mL/min/1.73 m^2^) between robot-assisted partial nephrectomy (RaPN) and laparoscopic cryoablation (LCA) during the postoperative follow-up period. The *p* value < 0.05 was regarded as statistically significant.

We used an estimated glomerular filtration rate (mL/min/1.73 m^2^) to represent the renal function outcomes in both groups. The change in renal function during the follow-up period is illustrated in Figure 2. Over its 5-year follow-up, the RaPN group demonstrated a significant decrease in renal function compared with baseline data (*p* < 0.001). Nevertheless, over its 5-year follow-up, the LCA group demonstrated nonsignificant renal function impairment (*p* = 0.085).

## 4. Discussion

When treating SRMs, selecting the optimal treatment option is crucial. The selection is based on several factors, such as patient morbidity, cancer control, renal function preservation, and treatment risks. The surgical management of SRMs has developed over the years, with an emphasis on nephron-sparing and minimally invasive procedures. RaPN and focal therapy application in SRM treatment has recently gained popularity [10], and thermal ablation is indicated in patients with multiple tumours and/or unable and/or unwilling to undergo more invasive surgery [11]. The 2019 guideline of European Association of Urology mentioned mixed study reports of oncological outcomes, perioperative outcomes, complication rates, new CKD developments, and other QoL measures compared with cryoablation with partial nephrectomy [12]. There is increasing evidence suggesting similar residual tumour rates and favourable disease control between the two approaches [13]. Some determined that cryoablation provides more effective renal preservation than does RaPN, along with decreased morbidity [2,6,14].

Preserving renal function is vital in the ageing population, because GFR demonstrates a significant decline after the age of 30 years [2]. Our findings demonstrate that renal function is better preserved in the LCA group, even with longer follow-up periods. The longer operative time and ischaemia time and increased blood loss in the RaPN group during the operation may play a role in the renal function decline [6,15]. Yoon et al. compared focal therapy versus robot-assisted partial nephrectomy for cT1 renal masses in a systematic review and meta-analysis and observed that focal therapy was more advantageous with regard to renal function preservation and bleeding [10].

Lin et al. compared outcomes for laparoscopic partial nephrectomy and LCA and observed that laparoscopic partial nephrectomy patients led to longer hospital stays (4 vs. 2 days) and more blood loss [16]. Zargar et al. released a collaborative review in 2016 considering cryoablation for SRMs [14]: Their overall rates of complications for renal cryoablation procedures were 7.8–20%, with most complications being minor (Clavien grade < 3). Tumour size, location, and medical comorbidities were critical factors related to the complications. In our study, the intraoperative and postoperative outcomes were similar in both groups, except for fever episode rates being higher in the RaPN group—which might be related to the longer operative time. The estimated blood loss was significantly higher in the RaPN group (*p* < 0.001) and manifested in decreases in renal function. Cryoablation is minimally invasive, with suitably low rates of complications [14]. A Cleveland Clinic study reported longer hospital stay (72 vs. 48 h; *p* < 0.0001) and higher intraoperative complication risk (20% vs. 12%; *p* = 0.015) for RaPN than for LCA [3,17]. A recent meta-analysis comparing LCA with minimally invasive partial nephrectomies demonstrated that the partial nephrectomies had a nearly doubled risk of both urological and nonurological complications [14]. Other studies on complications between these groups revealed higher conversion rates to nephrectomy in RaPN than in LCA (4.3% vs. 0%) [3].

The oncological outcomes of RCC treatment are substantial. Emara et al. compared RaPN and LCA for SRMs and reported a 3.6% local recurrence rate in the LCA group compared with a 0% rate in the RaPN group—despite follow-up time in the LCA group being longer [3,17]. Guilloteau et al. reported a higher recurrence rate for LCA than for RaPN (11% vs. 0%). However, the follow-up duration in the LCA group was also considerably longer (44.5 vs. 4.8 months) [14]. Nevertheless, recurrence rate is not the leading cause of mortality. In their series with one of the longest follow-up durations after LCA, Aron et al. reported the outcomes of 55 patients with biopsy-proven RCC, with a median follow-up of 93 months: the 5-year overall survival was 84% and 5-year cancer-specific survival and recurrence-free survival were 92% and 81%, respectively [14]. In our study, nearly all patient with tumour sized ≤4 or >4 cm did not demonstrate local recurrence, except for two patients with tumours sized >4 cm in the LCA group. No patient died of RCC during the follow-up period in either group. There was no significant difference in cancer-specific mortality or overall mortality between the RaPN and LCA groups in either tumour size groups. Yoon et al. reviewed and compared studies on RaPN and focal therapy (FT) for renal mass treatment: Tanagho et al. did not report a significant difference in 5-year cancer-specific survival rate (100% vs. 96.4%, *p* = 0.41) and overall survival rate (91.7% vs. 77.1%, *p* = 0.11). Kim et al. did not report a difference in cancer-related deaths (7.4% vs. 3.7%, *p* = 0.764); similarly, Caputo et al. reported that cancer-specific death (0.6% vs. 3.2%; *p* = 0.48) and overall mortality (3.1% vs. 19.3%, *p* = 0.155) did not differ significantly [10]. Several studies investigating cancer-specific survival and overall survival have revealed no differences between RaPN and FT—this agrees with our data [10]. Notably, matched studies for similar basic characteristics have revealed similar local recurrence rates for RaPN and FT [10]. FT has been reported to be the optimal treatment for SRMs [10]. Radical nephrectomy is the conventional treatment for a cT1b renal tumour; however, the interest in using partial nephrectomy or cryoablation treatment for selected patients with cT1b renal tumours has been growing [10,18]. Caputo et al. also reported a retrospective matched comparative analysis of patients with cT1b renal mass who underwent renal LCA, percutaneous cryoablation, or RaPN. The total postoperative complication rate was higher for RaPN than that for cryoablation (42% vs. 23%; *p* = 0.10) [19]. There was no significant difference in cancer-specific mortality (*p* = 0.5) or overall mortality (*p* = 0.15) between the cryoablation and partial nephrectomy groups [19]. Two studies by the Mayo group reported similar local tumour control rates among small (size < 3 cm) and large (size = 3–8.3 cm) renal masses, suggesting that tumour size is not a risk factor for tumour recurrence [14]. Here, the oncological outcomes and survival rate in the LCA group were comparable to the RaPN group for tumours sized ≤4 as well as >4 cm. In addition, no significant difference was observed in the complication rate between the two tumour size groups.

This study has several limitations. A major limitation is its retrospective and nonrandomised trial design with relatively small case numbers from a single centre. This study enrolled first cases of RaPN in our hospital, and the learning curve of this technique should be taken into account. Moreover, although its perioperative outcomes are promising, longer follow-up and further larger number studies are needed to confirm this novel finding.

## 5. Conclusions

In conclusion, both LCA and RaPN appear to be safe and effective for localised renal tumour treatment. LCA has potential benefits in reduced blood loss, shorter operative time, and slight renal function deterioration. Better quality evidence is needed to make informed conclusions on efficacy.

## Figures and Tables

**Table 1:** Demographic characteristics.

Characteristic		RaPN	LCA	*p*-Value
Case number, n		55	55	
Gender, % (n)				>0.99
	Males	52.7% (29)	52.7% (29)	
	Females	47.3% (26)	47.3% (26)	
Systemic diseases, % (n)				
	CAD	5.5% (3)	5.5% (3)	>0.99
	HTN	40.0% (22)	45.5% (25)	0.563
	DM	20.0% (11)	18.2% (10)	0.808
	CKD	18.2% (10)	23.6% (13)	0.482
	Other malignancy	16.4% (9)	14.5% (8)	0.792
Mean age, years		57.27 ± 13.28	59.44 ± 14.77	0.616
ASA score, % (n)				0.644
	1	0% (0)	0% (0)	
	2	76.4% (42)	80.0% (44)	
	3	23.6% (13)	20.0% (11)	
	4	0% (0)	0% (0)	
Mean BMI, kg/m^2^		25.29 ± 4.58	25.04 ± 4.23	0.245
Anesthesia, % (n)				-
	LA	0% (0)	0% (0)	
	GA	100% (55)	100% (55)	
Tumor size, cm		4.06 ± 2.01	3.86 ± 2.13	0.796
Clinical T stage, % (n)				
	T1a	60.0% (33)	58.2% (32)	0.846
	T1b	36.4 % (20)	40.0% (22)	0.695
	T2a	0 % (0)	0% (0)	-
	T2b	3.6% (2)	1.8% (1)	>0.99
Tumor side, % (n)				>0.99
	Left	45.5% (25)	45.5% (25)	
	Right	54.5% (30)	54.5% (30)	

RaPN, Robot-assisted laparoscopic partial nephrectomy. LCA, Laparoscopic cryoablation. CAD, Coronary artery disease. HTN, Hypertension. DM, Diabetes mellitus. CKD, Chronic kidney disease. ASA, American Society of Anesthesiologists. BMI, Body mass index (Kg/m^2^). LA, Local anesthesia. GA, General anesthesia. The *p* value < 0.05 was regarded as statistically significant.

**Table 2:** Intraoperative and postoperative outcomes.

Characteristic		RaPN (n = 55)	LCA (n = 55)	*p*-Value
Follow-up, month		33.20 ± 19.55	54.96 ± 34.59	<0.001 *
Approach, % (n)				
	Transperitoneal	43.6% (24)	3.6% (2)	<0.001 *
	Retroperitoneal	56.4% (31)	96.4% (53)	
Probe of cryoablation, n		-	2.54 ± 1.28	-
Robotic arms, n		3.30 ± 0.46	-	-
Operative time, min		267.45 ± 104.53	138.56 ± 45.28	<0.001 *
Warm ischemia time, min		23.70 ± 16.58	-	-
Console time, min		165.96 ± 70.35	-	-
LOS, day		6.11 ± 5.10	4.15 ± 2.71	0.239
Estimated blood loss, mL		300.56 ± 360.73	30.73 ± 50.31	<0.001 *
Preoperative Hb, g/dL		13.38 ± 1.71	13.22 ± 1.89	0.314
Change of Hb, g/dL		−1.59 ± 1.27	−1.11 ± 1.18	0.466
Preoperative Hct, %		40.32 ± 4.41	36.69 ± 5.34	0.272
Change of Hct, %		−5.10 ± 3.79	−3.67 ± 3.44	0.234
Intra-operative complication, % (n)				
	Open conversion	3.6% (2)	0% (0)	0.495
	Bowel injury	1.8% (1)	0% (0)	>0.99
Post-operative complication, % (n)				
	Blood transfusion	23.6% (13)	14.5% (8)	0.225
	Fever	21.8% (12)	5.5% (3)	0.012 *
	Subcutaneous emphysema	0% (0)	1.8% (1)	>0.99
	Pneumothorax	0% (0)	1.8% (1)	>0.99
	Hemorrhage need intervention	0% (0)	1.8% (1)	>0.99
Complications, % (n)				
	Minor, Clavien 1–2	0% (0)	5.5% (3)	0.243
	Major, Clavien 3–5	3.6% (2)	3.6% (2)	>0.99

RaPN, Robot-assisted laparoscopic partial nephrectomy. LCA, Laparoscopic cryoablation. LOS, Length of stay. Hb, Hemoglobin. Hct, Hematocrit. * The *p* value < 0.05 was regarded as statistically significant.

**Table 3:** Pathological outcomes.

Characteristic		RaPN (n = 55)	LCA (n = 55)	*p*-Value
RCC histology, % (n)		58.2% (32)	49.1% (27)	0.339
	Clear cell RCC, % (n)	45.5% (25)	32.7% (18)	0.171
	Papillary RCC, % (n)	0% (0)	1.8% (1)	>0.99
	Chromophobe RCC, % (n)	9.1% (5)	1.8% (1)	0.206
	MiT Family translocation RCC, % (n)	1.8% (1)	0% (0)	>0.99
	Mixed epithelial and stromal tumor, % (n)	0% (0)	0% (0)	-
	Unclassified, % (n)	1.8% (1)	12.7% (7)	0.06
Benign conditions, % (n)		41.8% (23)	49.1% (27)	0.444
	Oncocytoma, % (n)	3.6% (2)	5.5% (3)	>0.99
	AML, % (n)	32.7% (18)	16.4% (9)	0.046 *
	Cyst, % (n)	3.6% (2)	1.8% (1)	>0.99
	Inconclusive or Negative for malignancy, % (n)	0% (0)	20.0% (11)	<0.001 *

RaPN, Robot-assisted laparoscopic partial nephrectomy. LCA, Laparoscopic cryoablation. RCC, Renal cell carcinoma. AML, Angiomyolipoma. * The *p* value < 0.05 was regarded as statistically significant.

**Table 4:** Oncological outcomes in the patients with RCC according to the operative method (tumour size ≤ 4 cm, n = 38).

Characteristic	RaPN (n = 23)	LCA (n = 15)	*p*-Value
Positive surgical margins, % (n)	0% (0)	-	-
Local recurrence, % (n)	0% (0)	0% (0)	-
Metastasis, % (n)	0% (0)	0% (0)	-
De novo tumor, % (n)	0% (0)	0% (0)	-

RCC, Renal cell carcinoma. RaPN, Robot-assisted laparoscopic partial nephrectomy. LCA, Laparoscopic cryoablation. OS, Overall survival. CSS, Cancer-specific survival. The *p* value < 0.05 was regarded as statistically significant.

**Table 5:** Oncological outcomes in the patients with RCC according to the operative method (tumour size > 4 cm, n = 21).

Characteristic	RaPN (n = 9)	LCA (n = 12)	*p*-Value
Positive surgical margins, % (n)	11.1% (1)	-	-
Local recurrence, % (n)	0% (0)	16.7% (2)	0.486
Metastasis, % (n)	11.1% (1)	0% (0)	0.429
De novo tumour, % (n)	11.1% (1)	0% (0)	0.429

RCC, Renal cell carcinoma. RaPN, Robot-assisted laparoscopic partial nephrectomy. LCA, Laparoscopic cryoablation. OS, Overall survival. CSS, Cancer-specific survival. The *p* value < 0.05 was regarded as statistically significant.

## Data Availability

The data presented in this study are available on request from the corresponding author. The data are not publicly available due to legal restrictions imposed by government of Taiwan in relation to the “Personal Information Protection Act”, data cannot be made publicly available.

## Supplementary Materials

The following are available online at https://www.mdpi.com/article/10.3390/diagnostics11050759/s1, Figure S1: The overall survival rates in the patients with RCC (tumour size ≤ 4 cm, n = 38) during the postoperative follow-up period. One patient died of hepatitis B virus flare up and hepatic failure in the RaPN group after 42.1 months follow-up. One patient with hepatocellular carcinoma HCC died of spontaneous bacterial peritonitis in the LCA group after 13.8 months follow-up. The p value < 0.05 was regarded as statistically significant, Figure S2: The overall survival rates in the patients with RCC (tumour size > 4 cm, n = 21) during the postoperative follow-up period. One patient with history of HCC died of spontaneous bacterial peritonitis and hepatic encephalopathy in the LCA group after 56.8 months follow-up. The p value < 0.05 was regarded as statistically significant, Table S1: Tumour characteristics: RENAL nephrometric scores of 110 patients with renal tumour.
