# Supplementary material for: Comparison of Robot-Assisted Laparoscopic Partial Nephrectomy with Laparoscopic Cryoablation in the Treatment of Localised Renal Tumours: A Propensity Score-Matched Comparison of Long-Term Outcomes
Source: Diagnostics (Basel). 2021 Apr 23;11(5):759. doi: 10.3390/diagnostics11050759 (PMC8146293; doi:10.3390/diagnostics11050759)
Supplement: Supplementary file 1 [file diagnostics-11-00759-s001.zip › diagnostics-1145755-supplementary.pdf]

diagnostics

Supplementary File

Comparison of Robot-Assisted Laparoscopic Partial Nephrectomy with Laparoscopic Cryoablation in the Treatment of Localised Renal Tumours: A Propensity Score-Matched Comparison of Long-Term Outcomes

Hui-Ying Liu [1], Chih Hsiung Kang [1], Hung-Jen Wang [1], Chien Hsu Chen [1], Hao Lun Luo [1,2], Yen-Ta Chen [1], Yuan-Tso Cheng [1] and Po-Hui Chiang [1,2*]

[1] Department of Urology, Kaohsiung Chang Gung Memorial Hospital and Chang Gung University College of Medicine, Kaohsiung 833, Taiwan; ying1011@cgmh.org.tw (H.-Y.L.); chkang5801@gmail.com (C.H.K.); hujewang@gmail.com (H.-J.W.); u8601062@cgmh.org.tw (C.H.C.); alesy@cgmh.org.tw (H.L.L.); adam@cgmh.org.tw (Y.-T.C.); ytcheng@cgmh.org.tw (Y.-T.C.)
[2] Graduate Institute of Medicine, College of Medicine, Kaohsiung Medical University, Kaohsiung 83301, Taiwan
* Correspondence: cphtem@yahoo.com.tw; Tel.: +88-677-317-123 (ext. 8094)

Citation: Liu, H.-Y.; Kang, C.H.; Wang, H.-J.; Chen, C.H.; Luo, H.L.; Chen, Y.-T.; Cheng, Y.-T.; Chiang, P.-H. Comparison of Robot-Assisted Laparoscopic Partial Nephrectomy with Laparoscopic Cryoablation in the Treatment of Localised Renal Tumours: A Propensity Score-Matched Comparison of Long-Term Outcome. **2021**, *11*, 759. https://doi.org/10.3390/diagnostics11050759

Academic Editor: Dil Sahali

Received: 27 February 2021
Accepted: 19 April 2021
Published: 23 April 2021

Publisher's Note: MDPI stays neutral with regard to jurisdictional claims in published maps and institutional affiliations.

Copyright: © 2021 by the authors. Licensee MDPI, Basel, Switzerland. This article is an open access article distributed under the terms and conditions of the Creative Commons Attribution (CC BY) license (http://creativecommons.org/licenses/by/4.0/).

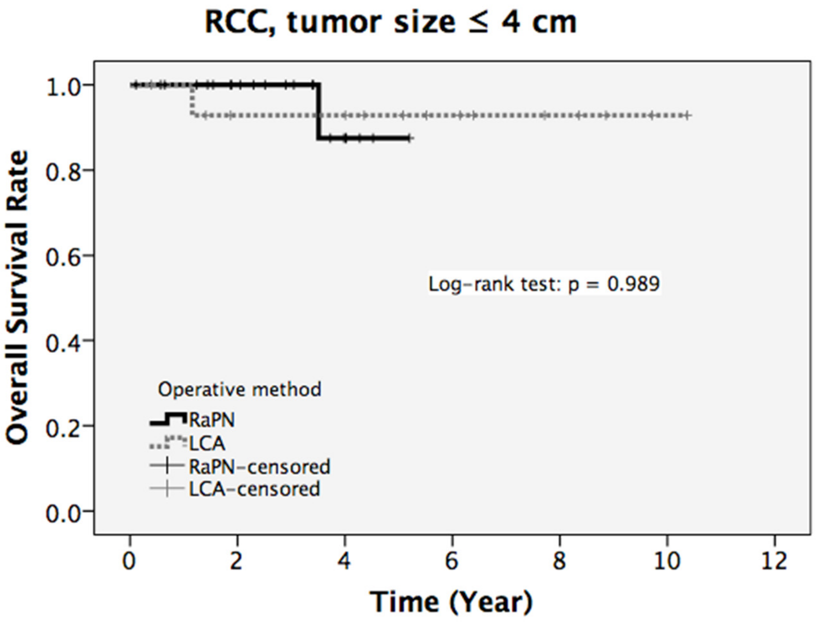

Figure S1. The overall survival rates in the patients with RCC (tumour size ≤ 4 cm, n = 38) during the postoperative follow-up period. One patient died of hepatitis B virus flare up and hepatic failure in the RaPN group after 42.1 months' follow-up. One patient with hepatocellular carcinoma HCC died of spontaneous bacterial peritonitis in the LCA group after 13.8 months follow-up. The p value < 0.05 was regarded as statistically significant.

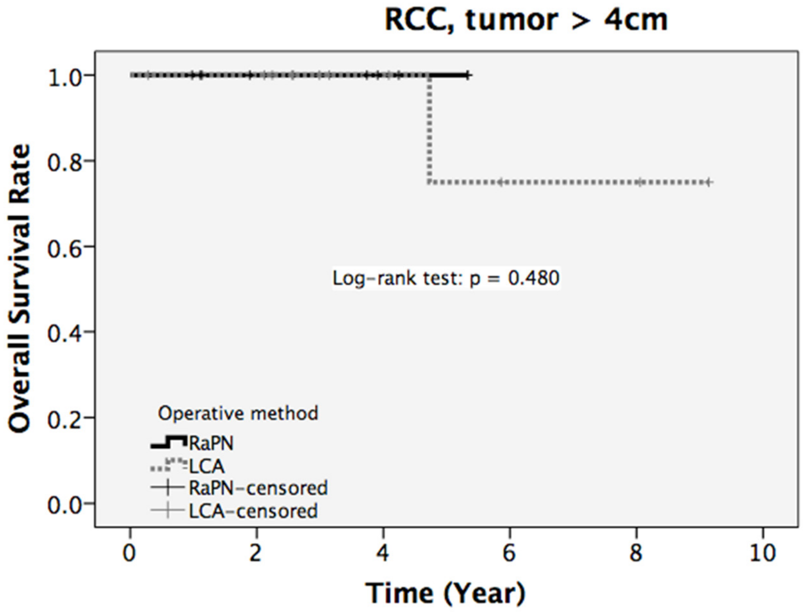

Figure S2. The overall survival rates in the patients with RCC (tumour size > 4 cm, n = 21) during the postoperative follow-up period. One patient with history of HCC died of spontaneous bacterial peritonitis and hepatic encephalopathy in the LCA group after 56.8 months' follow-up. The *p* value < 0.05 was regarded as statistically significant.

Table S1. Tumour characteristics: RENAL nephrometric scores of 110 patients with renal tumour.

Characteristic	RaPN (n = 55)	LCA (n = 55)	*p*-value
**R (size, cm), % (n)**			>0.99
**≤ 4 (1 point)**	58.2% (32)	58.2% (32)	
**4 but < 7 (2 points)**	38.2% (21)	40.0% (22)	
**≥ 7 (3 points)**	3.6% (2)	1.8% (1)	
**E (exophicity), % (n)**			0.192
**≥ 50% (1 point)**	30.9% (17)	16.4% (9)	
**< 50% (2 points)**	30.9% (17)	40.0% (22)	
**Entirely endophytic (3 points)**	38.2% (21)	43.6% (24)	
**N (nearness to collecting system, mm), % (n)**			0.483
**≥ 7 (1 point)**	43.6% (24)	38.2% (21)	
**> 4 but < 7 (2 points)**	20.0% (11)	14.5% (8)	
**≤ 4 (3 points)**	36.4% (20)	47.3% (26)	
**A (anterior vs posterior), % (n)**			0.807
**Anterior**	30.9% (17)	32.7% (18)	
**Posterior**	40.0% (22)	45.5% (25)	
**Lateral**	21.8% (12)	14.5% (8)	
**Inconclusive**	7.3% (4)	7.3% (4)	
**L (location relative to polar line), % (n)**			0.821
**Above or below polar line (1 point)**	30.9% (17)	34.5% (19)	
**Cross polar line (2 points)**	34.5% (19)	29.1% (16)	
**Between polar line (3 points)**	34.5% (19)	36.4% (20)	
**Total RENAL nephrometric score**	7.49 ± 1.86	7.82 ± 1.93	0.535
**Low (4–6), % (n)**	30.9% (17)	32.7% (18)	0.838
**Intermediate (7–9), % (n)**	49.1% (27)	41.8% (23)	0.444
**High (10–12), % (n)**	20.0% (11)	25.9% (14)	0.462

RaPN, Robot-assisted laparoscopic partial nephrectomy. LCA, Laparoscopic cryoablation. *The p value < 0.05 was regarded as statistically significant.
